# Real-world outcomes of observation and treatment in diabetic macular edema with very good visual acuity: the OBTAIN study

**DOI:** 10.1007/s00592-019-01310-z

**Published:** 2019-03-22

**Authors:** Catharina Busch, Samantha Fraser-Bell, Dinah Zur, Patricio J. Rodríguez-Valdés, Zafer Cebeci, Marco Lupidi, Adrian T. Fung, Pierre-Henry Gabrielle, Ermete Giancipoli, Voraporn Chaikitmongkol, Mali Okada, Inês Laíns, Ana Rita Santos, Paradee Kunavisarut, Anna Sala-Puigdollers, Jay Chhablani, Malgorzata Ozimek, Assaf Hilely, Jan Darius Unterlauft, Anat Loewenstein, Matias Iglicki, Matus Rehak, For the International Retina Group

**Affiliations:** 1 Department of Ophthalmology, University Hospital Leipzig, Liebigstr. 10-14, 04103 Leipzig, Germany; 2 Department of Ophthalmology, Sydney University, Sydney, Australia; 3 Division of Ophthalmology, Tel Aviv Sourasky Medical Center, Tel Aviv, Israel; 4 Sackler Faculty of Medicine, Tel Aviv University, Tel Aviv, Israel; 5 Instituto de Oftalmología y Ciencias Visuales, Escuela de Medicina, Tecnológico de Monterrey, Monterrey, Mexico; 6 Istanbul Faculty of Medicine, Department of Ophthalmology, Istanbul University, Istanbul, Turkey; 7 Department of Biomedical and Surgical Sciences, Section of Ophthalmology, University of Perugia, Perugia, Italy; 8 Department of Ophthalmology, Westmead Hospital, Sydney, Australia; 9 Faculty of Medicine and Health Sciences, Macquarie University Hospital, Sydney, Australia; 10 Ophthalmology Department, Dijon University Hospital, Dijon, France; 11 Center for Taste and Feeding Behaviour, INRA, UMR1324, Dijon, France; 12 Department of Surgical, Microsurgical and Medical Sciences, Eye Clinic, University of Sassari, Sassari, Italy; 13 Department of Biomedical Sciences, University of Sassari, Sassari, Italy; 14 Retina Division, Department of Ophthalmology, Faculty of Medicine, Chiang Mai University, Chiang Mai, Thailand; 15 Royal Victorian Eye and Ear Hospital, Melbourne, VIC Australia; 16 Faculty of Medicine, University of Coimbra, Coimbra, Portugal; 17 Association for Innovation and Biomedical Research on Light and Image, Coimbra, Portugal; 18 Massachusetts Eye and Ear, Harvard Medical School, Boston, USA; 19 Department of Orthoptics, Superior School of Health, Polytechnic of Porto, Porto, Portugal; 20 Institut Clínic d’Oftalmologia (ICOF), Hospital Clínic de Barcelona, Barcelona, Spain; 21 L.V. Prasad Eye Institute, Banjara Hills, Hyderabad, India; 22 Department of General Ophthalmology and Pediatric Ophthalmology Service, Medical University in Lublin, Lublin, Poland; 23 Incumbent, Sydney A. Fox chair in Ophthalmology, Tel Aviv University, Tel Aviv, Israel; 24 Private Retina Service, University of Buenos Aires, Buenos Aires, Argentina

**Keywords:** Diabetic macular edema, Good visual acuity, Observation, Anti-VEGF therapy, Intravitreal therapy, Macular laser

## Abstract

**Aims:**

To describe and compare the functional and anatomical outcomes of untreated and treated diabetic macular edema (DME) in eyes with very good baseline visual acuity (VA) in a real-world setting.

**Methods:**

A 12-month, retrospective, multicenter, observational cohort study, including DME patients with baseline visual acuity (VA) ≤ 0.1 logMAR (≥ 20/25 Snellen) and central subfield thickness (CST) > 250 µm with intra- and/or subretinal fluid seen on optical coherence tomography.

**Results:**

A total of 249 eyes were included, of which 155 were treated and 94 were non-treated during follow-up. Most eyes maintained vision (VA gain or VA loss < 5 letters) at 12 months (treated: 58.1%; non-treated: 73.4%). In non-treated eyes with stable VA within the first 6 months, VA was maintained throughout the follow-up in most cases (86.3%). In non-treated eyes with VA loss ≥ 5 letters within 6 months (36.7%), further observation led to worse visual outcome than treatment (− 4.2 vs. − 7.8 letters, *p* = 0.013). In eyes in which treatment was initiated at baseline (*n* = 102), treatment with 8–12 anti-VEGF injections led to better visual outcome compared to treatment with less injections (− 0.3 ± 3.6 letters vs. − 3.8 ± 6.2 letters, *p* = 0.003).

**Conclusion:**

In a real-world setting, the majority of DME patients with very good VA maintained vision at 12 months, regardless of whether the DME was treated or not. This study supports close observation of eyes with DME and very good VA with consideration of treatment when a one line drop in vision is observed.

## Introduction

Diabetic macular edema (DME) is the main cause of vision loss in diabetic patients affecting around 21 million people worldwide [1, 2]. Several treatment regimens, including macular laser, intravitreal anti-VEGF injections, intravitreal triamcinolone acetonide, and dexamethasone (DEX) intravitreal implant have been shown to be effective for DME in randomized controlled trials (RCT) [3–12]. However, RCTs excluded eyes with very good vision so far, and hence little is known about the visual prognosis of such eyes with or without treatment. Protocol V by DRCR.net is the first RCT on central-involved DME and good visual acuity comparing prompt focal/grid photocoagulation, observation and prompt anti-VEGF therapy [13]. This trial is currently ongoing and the first results are awaited.

The main purpose of this study was to assess the functional and anatomical outcome of patients with DME with very good baseline visual acuity in a real-world setting.

## Methods

This is a retrospective, international, multicenter, observational cohort study comprising 16 study sites. Institutional review board (IRB) approval was obtained through the individual IRBs at the participating institutes for a retrospective consecutive chart review. This research adhered to the tenets of the Declaration of Helsinki.

### Study participants

Medical records of patients from January 1st, 2010, to June 30th, 2017 with a diagnosis of DME were reviewed. The following were set as inclusion criteria, with all criteria being met: (1) age 18 years or older; (2) type 1 or 2 diabetes mellitus; study eye with (3) center-involving DME (DME defined by retinal thickness of > 250 µm in the central subfield thickness (CST)) and intra ± subretinal fluid on spectral-domain optical coherence tomography (SD-OCT). (4) Best corrected visual acuity ≤ 0.1 logMAR (≥ 0.8 decimal acuity, ≥ 20/25 or ≥ 80 EDTRS letters).

Exclusion criteria were (1) concomitant ocular disease that could cause macular edema (including choroidal neovascularization from any cause, retinal vein occlusion, uveitis and recent intraocular surgery); (2) any concomitant ocular or neurological condition that could affect vision except cataract; (3) laser panretinal photocoagulation (PRP) < 6 months prior to baseline; (4) intravitreal therapy < 3 months prior to study inclusion; and (5) intravitreal therapy during follow-up for proliferative diabetic retinopathy (PDR).

### Data collection

For eligible patients, the following data were collected from their medical charts: demographic data (i.e., age, sex); duration of diabetes; stage of diabetic retinopathy [non-proliferative (NPDR) or PDR]; previous DME treatments (macular laser, intravitreal anti-VEGF injections, triamcinolone acetonide, DEX implant), previous laser PRP; lens status at baseline and 12 months; VA and CST at baseline, 3, 6, 9 and 12 months; and further treatment during follow-up (including macular laser, intravitreal anti-VEGF injections, triamcinolone acetonide, and DEX implant), laser PRP, and cataract surgery.

### Outcome measures

Main outcome measures were the mean change in VA and CST from baseline to month 12. Secondary outcome measures included the mean change in VA and CST from baseline to month 6, the proportion of eyes which maintained vision (VA loss < 5 letters or VA gain), VA loss ≥ 5 letters, ≥ 10 letters, ≥ 15 letters, VA of ≥ 0.2 logMAR (≤ 75 letters, ≤ 20/32 Snellen equivalent) and VA of ≥ 0.3 logMAR (≤ 70 letters, ≤ 20/40 Snellen equivalent) at 12 months.

### OCT analysis

All eyes were imaged with SD-OCT (Heidelberg Spectralis, Heidelberg, Germany; Optovue Avanti, Fremont, USA; Topcon 3D OCT-2000, Tokyo; Japan; or Cirrus, Zeiss, Oberkochen, Germany, Canon-OCT HS100, Tokyo, Japan). Quantitative assessment of DME-included CST calculated automatically by the instrument. Additionally, for all study participants the horizontal B-scans encompassing the fovea were exported. These images were graded for any disruption to the ellipsoid zone (EZ) by three independent and masked graders (CB, MI, MR).

### Statistical analysis

Variables are expressed as mean ± standard deviation (SD). To control for the correlated nature of our data, we used a generalized estimating equations (GEE) procedure. Differences in VA and CST between baseline and month 6 or month 12 were analyzed by univariable linear regression. Difference in outcome measures between the subgroups were assessed by including the following confounding baseline variables: (1) age, (2) gender, (3) stage of diabetic retinopathy (NPDR vs. PDR), (4) duration of diabetes, (5) EZ disruption at baseline, (6) lens status at baseline and (7) after 12 months, (8) treatment naivety, (9) conduction of PRP during follow-up, and (10) baseline VA (for VA outcomes) and baseline CST (for CST outcomes). Variables with *p* ≤ 0.15 in the univariable analysis were included in the final GEE model. A backward selection procedure was applied that retained only those variables with *p* < 0.05. For continuous outcome variables, a linear regression model and for a binary outcome a logistic regression model was applied. Markov chain Monte Carlo multiple imputation procedure with 100 run imputations was used to impute missing data. Statistical analysis was performed with SPSS Statistics 22 (IBM, Armonk, NY, USA).

## Results

The study included 249 eyes from 210 patients. Demographic and baseline characteristics are shown in Table 1. In the overall cohort, mean baseline VA was 0.06 ± 0.05 logMAR (82 letters, 20/25 Snellen equivalent) and mean baseline CST was 355.5 ± 77.3 µm (Table 2).

**Table 1:** Demographic and baseline characteristics

	Overall cohort (*n* = 249)	Eyes treated during F/U (*n* = 155)	Eyes not treated during F/U (*n* = 94)	*p* value*
Age, years, mean (SD)	60.1 (10.7)	58.1 (10.7)	63.5 (10.0)	< 0.001
Male, *n* (%)	145 (58.2)	63 (40.6)	53 (56.4)	0.660
HbA1c, %, mean (SD)	7.8 (1.5) *n* = 185	7.7 (1.3) *n* = 115	7.9 (1.7) *n* = 70	0.417
Duration of diabetes, months, mean (SD)	160.2 (124.7) *n* = 221	160.6 (122.2) *n* = 136	159.8 (129.4) *n* = 85	0.964
Proliferative diabetic retinopathy, *n* (%)	57 (22.9)	47 (30.3)	10 (10.6)	0.001
Type 1 diabetes, *n* (%)	22/247 (8.8)	17/153 (11.1)	5 (5.3)	0.190
Known comorbidities, *n* (%)				
None	41/237 (17.3)	33/145 (22.8)	8/92 (8.7)	0.016
Hypertension	185/239 (77.4)	101/146 (69.2)	84/93 (90.3)	0.001
Dyslipidemia	75/223 (33.6)	46/137 (33.6)	29/86 (33.7)	0.983
Diabetes therapy, *n* (%)				
Insulin	131/226 (58.0)	84/137 (61.3)	47/89 (52.8)	0.229
Metformin	100/216 (46.3)	54/127 (42.5)	46/89 (51.7)	0.204
Other oral antidiabetics	54/216 (25.0)	36/127 (28.3)	18/89 (20.2)	0.214
Other pharmacological therapies, *n* (%)				
Antiaggregant	59/226 (26.1)	36/137 (26.3)	23/89 (25.8)	0.941
Statins	67/226 (29.6)	40/137 (29.2)	27/89 (30.3)	0.859
ACE inhibitors	57/213 (26.8)	32/126 (25.4)	25/87 (28.7)	0.599
Sartanics	42/213 (19.7)	21/126 (16.7)	21/87 (24.1)	0.201
Beta blockers	46/213 (21.6)	24/126 (15.1)	22/87 (25.3)	0.246
Calcium antagonists	33/213 (15.5)	19/126 (15.1)	14/87 (16.1)	0.845
Diuretics	38/213 (17.8)	19/126 (15.1)	19/87 (21.8)	0.222
Treatment-naïve DME, *n* (%)	186 (74.7)	109 (70.3)	77 (81.9)	0.040
Prior macular laser, *n* (%)	38 (15.3)	24 (15.5)	14 (14.9)	0.899
Prior anti-VEGF therapy, *n* (%)	43 (17.3)	34 (21.9)	9 (9.6)	0.015
No. of prior anti-VEGF injections, mean (SD)	5.2 (3.2)	5.4 (3.4)	4.8 (2.8)	0.588
Prior therapy with IVTA, *n* (%)	3 (1.2)	2 (1.3)	1 (1.1)	0.874
Prior therapy with DEX implant, *n* (%)	1 (0.4)	0 (0)	1 (1.1)	–
Pseudophakia, *n* (%)	42 (16.9)	29 (18.7)	13 (13.8)	0.343
Prior PRP, *n* (%)	66 (26.5)	48 (31.0)	18 (19.1)	0.059
EZ disruption, *n* (%)	56/234 (23.9)	44/143 (28.4)	12/91 (13.2)	0.003

*DEX* dexamethasone, *DME* diabetic macular edema, *EZ* ellipsoid zone, *HbA1c* hemoglobin A1c, *IVTA* intravitreal triamcinolone acetonide, *PRP* panretinal photocoagulation, *SD* standard deviation, *VEGF* vascular endothelial growth factor

*p value for difference between treated and observed eyes, tested by univariable regression analysis

**Table 2:** Study outcomes

	Baseline VA, logMAR, mean (SD)	VA change 6M, letters, mean (SD)	VA change 12M, letters, mean (SD)	Baseline CST, µm, mean (SD)	CST change 6M, µm, mean (SD)	CST change 12M, µm, mean (SD)
All eyes, *n* = 249	0.06 (0.05)	− 2.5 (6.2)	− 2.8 (5.8)	355.5 (77.3)	− 13.2 (72.6)	− 19.9 (88.5)
All eyes observed over 12M, *n* = 94	0.05 (0.06)	− 0.6 (3.2)	− 1.8 (5.6)	315.6 (34.9)	+ 0.3 (34.1)	+ 11.3 (58.8)
All eyes treated over 12M, *n* = 155	0.06 (0.04)	− 3.6 (7.3)	− 3.4 (5.8)	379.6 (85.7)	− 21.3 (87.2)	− 38.9 (97.7)
All eyes observed over 12M with stable VA* within first 6M, *n* = 73	0.05 (0.05)	+ 0.5 (2.3)	− 0.1 (3.8)	317.4 (33.7)	+ 0.4 (36.5)	+ 5.9 (58.4)
Eyes observed at baseline and with VA loss ≥ 5 letters within 6M						
Further observed, *n* = 21	0.06 (0.07)	− 4.6 (2.9)	− 7.8 (6.9)	309.5 (39.1)	− 0.1 (24.7)	+ 30.3 (58.0)
Treated, *n* = 33	0.06 (0.05)	− 6.2 (5.5)	− 4.1 (5.6)	382.9 (88.8)	+ 11.4 (68.5)	− 37.4 (70.3)
Eyes treated at baseline						
1–4 anti-VEGF injections, *n* = 34	0.06 (0.05)	− 2.7 (5.9)	− 3.5 (1.3)	382.4 (88.3)	− 34.1 (86.5)	− 48.8 (90.8)
5–7 anti-VEGF injections, *n* = 29	0.07 (0.04)	− 2.8 (5.9)	− 4.2 (5.9)	404.6 (97.8)	− 31.6 (111.4)	− 32.8 (146.6)
8–12 anti-VEGF injections, *n* = 17	0.07 (0.04)	− 1.6 (4.2)	− 0.3 (3.6)	397.4 (96.0)	− 42.5 (123.6)	− 85.9 (102.0)
All eyes treated with						
Anti-VEGF therapy only, *n* = 107	0.06 (0.04)	− 2.9 (5.5)	− 3.2 (5.6)	382.6 (86.7)	− 24.5 (93.1)	− 47.8 (101.4)
Anti-VEGF + Macular laser, *n* = 21	0.07 (0.04)	− 4.8 (6.9)	− 2.7 (5.6)	370.4 (78.4)	+ 9.0 (83.4)	− 21.5 (90.9)
Macular laser only, *n* = 18	0.04 (0.04)	− 6.8 (14.1)	− 4.6 (6.1)	357.7 (100.8)	− 17.9 (43.3)	+ 4.4 (78.2)

Data were missing as follows: VA at 6 months: 7.6%, CST at 6 months: 11.2%, VA at 12 months: 6.8%, CST at 12 months: 9.6%. Missing data were imputed by Markov chain Monte Carlo multiple imputation procedure with 100 run imputations

*CST* central subfield thickness, *M* months, *SD* standard deviation, *VA* visual acuity, *VEGF* vascular endothelial growth factor

*VA loss ≤ 4 letters or VA gain

The majority of eyes were treatment naïve (186/249, 74.7%). One quarter (63 eyes) had received DME treatment prior to inclusion in the study; including macular laser in 38 eyes (15.3%), anti-VEGF therapy in 43 eyes (17.3%), intravitreal triamcinolone acetonide in 3 eyes (1.2%) and DEX Implant in 1 eye (0.4%).

Over the 12 months of follow-up, 94 eyes (37.7%) were non-treated (never treated), and 155 eyes (62.2%) received treatment. Types of DME treatment undertaken during the study period is shown in Table 3. The cohort receiving treatment during the study period showed signs of a more severe disease with increased proportion of PDR, were more likely to have been previously treated and more likely to have EZ disruption on OCT imaging at baseline (Table 1).

**Table 3:** Treatment characteristics within 12-month follow-up

Eyes treated, *n* (%)	155 (62.2)
Macular laser, *n* (%)	39 (25.1)
Anti-VEGF therapy, *n* (%)	136 (88.9)
Anti-VEGF therapy only, *n* (%)	107 (69.9)
No. of anti-VEGF injections, mean (SD)	4.7 (2.6)
No. of ranibizumab injections, mean (SD)	3.0 (2.7)
No. of aflibercept injections, mean (SD)	0.9 (2.2)
No. of bevacizumab injections, mean (SD)	0.8 (2.0)
Triamcinolone acetonide, *n* (%)	1 (0.7)
No. of triamcinolone acetonide injections, mean (SD)	1.0 (0.0)
DEX implant, *n* (%)	8 (5.2)
No. of DEX implants, mean (SD)	1.0 (0.0)
Additional treatment, *n* (%)	
Panretinal photocoagulation	32/249 (12.9)
Conduction of cataract surgery	12/207 (5.8)

*DEX* dexamethasone, *SD* standard deviation, *VEGF* vascular endothelial growth factor

### Functional and anatomical outcomes

Most eyes maintained vision (VA gain or VA loss < 5 letters) at 12 months (treated eyes: 58.1%; non-treated eyes: 73.4%; Table 4). Mean change in VA at 12 months in non-treated eyes was − 1.8 ± 5.6 letters and − 3.4 ± 5.8 letters in treated eyes (Table 2). A VA loss of ≥ 5 letters was seen in 26.6% (25/94 eyes) of the non-treated cohort, and in 41.9% (65/155 eyes) of the treated cohort.

**Table 4:** Proportion of visual acuity outcomes at 12 months

	VA loss < 5 letters or VA gain, *n* (%)	VA loss ≥ 5 letters, *n* (%)	VA loss ≥ 10 letters, *n* (%)	VA loss ≥ 15 letters, *n* (%)	VA ≤ 20/32 Snellen equivalent, *n* (%)	VA ≤ 20/40 Snellen equivalent, *n* (%)
All eyes, *n* = 249	159 (63.9)	90 (36.1)	38 (15.3)	17 (6.8)	70 (28.1)	30 (12.0)
All eyes observed over 12M, *n* = 94	69 (73.4)	25 (26.6)	8 (8.5)	5 (5.3)	20 (21.3)	7 (7.4)
All eyes treated over 12M, *n* = 155	90 (58.1)	65 (41.9)	30 (19.4)	12 (7.7)	50 (32.3)	23 (14.8)
All eyes observed over 12M with stable VA* within first 6M, *n* = 73	63 (86.3)	10 (13.7)	1 (1.4)	0 (0)	7 (9.6)	1 (1.4)
Eyes observed and with VA loss ≥ 5 letters within 6M						
Further observed, *n* = 21	6 (28.6)	15 (71.4)	7 (33.3)	5 (23.8)	13 (61.9)	6 (28.6)
Treated, *n* = 33	17 (51.5)	16 (48.5)	6 (18.2)	3 (9.1)	12 (36.4)	4 (12.1)
Eyes treated at baseline						
1–7 anti-VEGF injections, *n* = 63	34 (54.0)	29 (46.0)	17 (27.0)	6 (9.5)	24 (38.1)	13 (20.6)
8–12 anti-VEGF injections, *n* = 17	13 (76.5)	4 (23.5)	0 (0)	0 (0)	4 (23.5)	0 (0)

*M* months, *VA* visual acuity, *VEGF* vascular endothelial growth factor

*VA loss ≤ 4 letters or VA gain

There was no clinical relevant change in CST at 12 months compared to baseline in non-treated eyes (+ 11.3 ± 58.8 µm, *p* = 0.06). However, at 12 months CST was reduced in eyes that were treated (− 38.9 ± 97.7 µm, *p* < 0.001; Table 2).

### Eyes non-treated at baseline

At the study baseline, treatment was commenced for 102 eyes (41.0%) whereas the other 147 eyes (59.0%) were initially non-treated.

In non-treated eyes with stable VA within the first 6 months, VA was maintained throughout the follow-up in most cases without any treatment (86.3%, Table 4). Only 1 eye dropped ≥ 10 letters (1.4%). In less than 10% (9.6% or 7/73 eyes) VA dropped to ≥ 0.2 logMAR (≤ 75 letters, ≤ 20/32 Snellen equivalent) at 12 months.

**Fig. 1:**
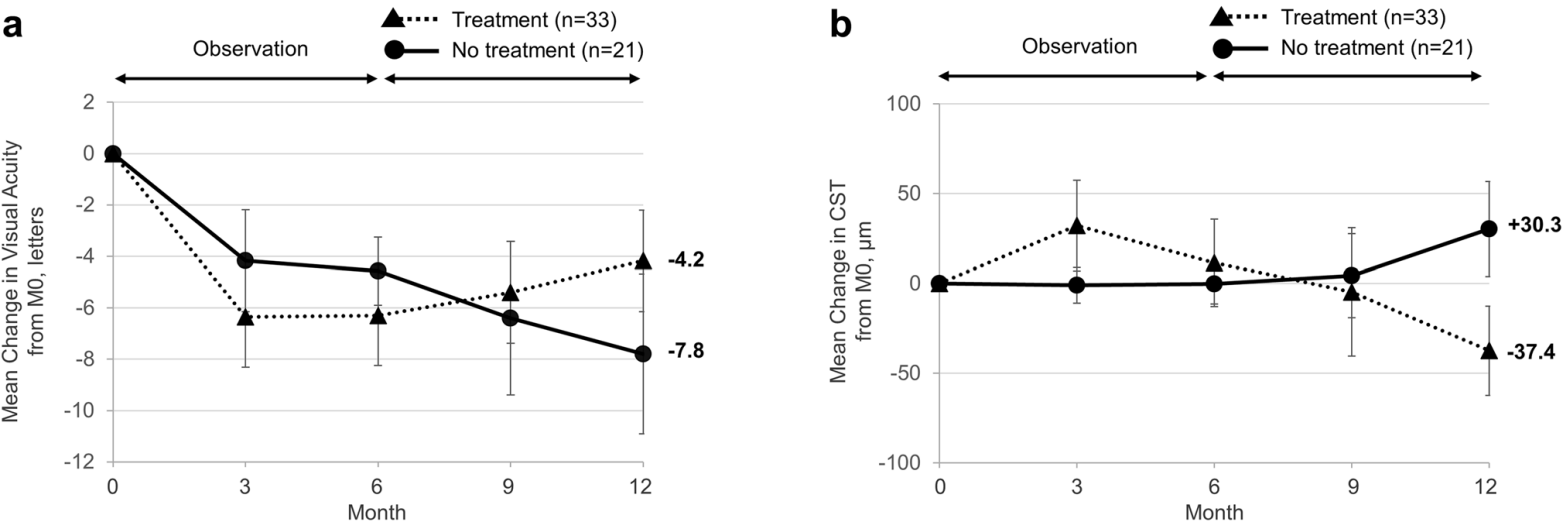
Mean change in visual acuity (**a**) and central subfield thickness (**b**, CST) over 12-month follow-up in eyes that were initially non-treated experiencing a VA loss ≥ 5 letters. Data are mean ± 95% confidence interval. *M0* baseline, month 0

If a VA loss ≥ 5 letters within 6 months occured (36.7%), further observation led to worse visual outcome than treatment (− 4.2 vs. − 7.8 letters, *p* = 0.013). Despite this VA loss within the first 6 months, 21 eyes (38.9%) continued to be non-treated over the study period. Those eyes experienced on average a small, but worse functional and anatomical outcome at 12 months than the 33 eyes (61.1%) that were treated after experiencing reduction in VA (VA change at 12 months: − 7.8 ± 6.9 letters vs. − 4.1 ± 5.6 letters, *p* = 0.013, multivariable analysis; CST change at 12 months: +30.3 ± 58.0 µm vs. − 37.4 ± 70.3 µm, *p* < 0.001, multivariable analysis; Fig. 1). Furthermore, treated eyes tended to be less likely to present with persistent VA loss of ≥ 5 letters at month 12 compared to eyes that were further observed after experiencing VA loss (48.5% vs. 71.4%, *p* = 0.100, multivariable analysis; Table 4).

### Eyes treated at baseline

Of the 102 eyes, in which treatment was initiated at baseline, 80 received anti-VEGF therapy with or without macular laser during the 12-month follow-up period.

**Fig. 2:**
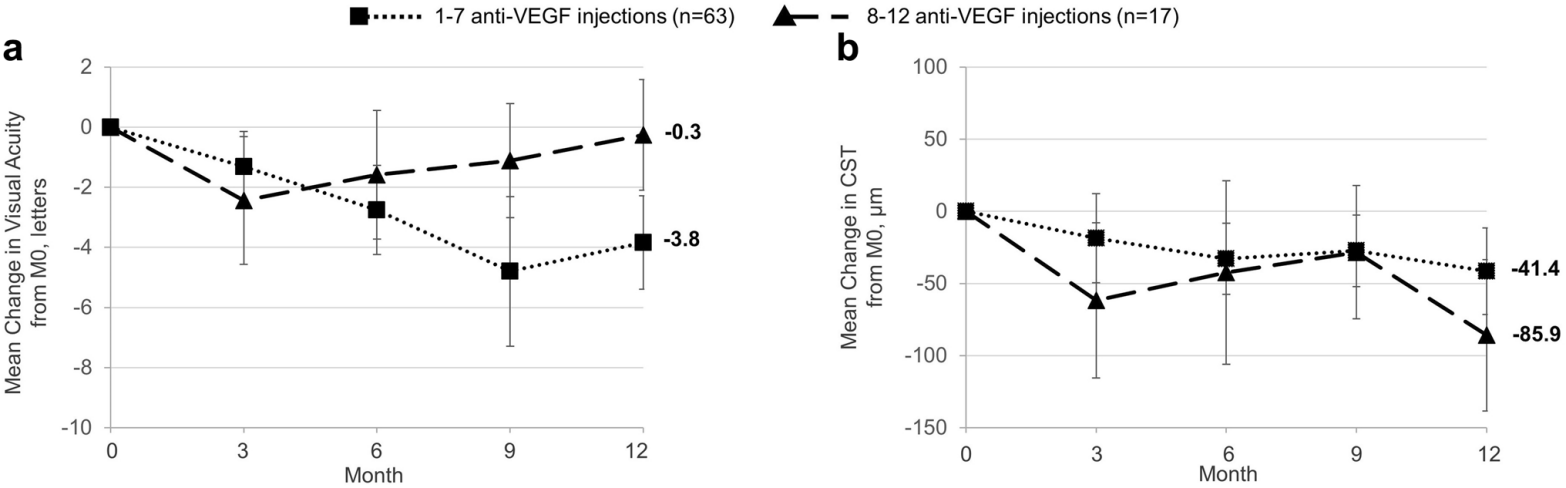
Mean change in visual acuity (**a**) and central subfield thickness (**b**, CST) over 12-month follow-up in eyes stratified for number of anti-VEGF injections. Data are mean ± 95% confidence interval. *VEGF* vascular endothelial growth factor, *M0* baseline, month 0

The combination of anti-VEGF + macular laser was not superior to anti-VEGF therapy only (VA change at 12 months: *p* = 0.683, CST change at 12 months: *p* = 0.227, univariable analysis; Table 2). Macular laser alone tended to lead to worse outcomes compared to intravitreal therapy (Table 2). Eyes that received 8–12 anti-VEGF injections on average showed a significantly better visual outcome compared to those that received 1–7 injections (VA change at 12 months: − 0.3 ± 3.6 letters vs. − 3.8 ± 6.2 letters, *p* = 0.003, multivariable analysis; Table 2; Fig. 2). There was a corresponding greater reduction in CST at 12 months but this was not statistically significant (CST change at 12 months: − 85.9 ± 102.0 µm vs. − 41.4 ± 119.0 µm, *p* = 0.068, multivariable analysis; Table 2; Fig. 2).

## Discussion

To our best knowledge, data on the real-world outcome of DME patients and very good baseline visual acuity have not been published. Previous RCTs and real-world studies did not include or report on DME eyes with baseline VA better than 78 letters [3–11, 14, 15]. Our study reveals that both non-treated and treated DME patients with very good visual acuity on average maintained very good vision after 12 months in a real-world setting. Untreated eyes without significant VA loss in the early observation phase maintained stable VA during the follow-up. However, in case of a significant VA loss under observation, treatment of those eyes led to better outcomes. In the treated cohort, intensive anti-VEGF treatment led to better functional and anatomical outcomes than less intense treatment. However, benefit reached by intensive treatment was small.

Kwon et al. reported on the natural course of DME by examining eyes with mild DME (CST 250–300 µm), but with worse VA [0.32 logMAR (20/50 Snellen equivalent) vs. 0.05 logMAR (20/25 Snellen equivalent)] than our cohort [16]. Similar to our cohort, VA acuity changes were small and a small but non-significant increase in CST in untreated DME eyes was observed [16]. Eyes that were never treated in our study on average maintained good visual acuity over the follow-up, raising the question if treatment should be considered in eyes with very good visual acuity. When eyes were non-treated and presented with a stable VA throughout the first 6 months, most eyes (86.3%) maintained VA over the whole study period. A relevant VA loss (≥ 5 letters) within the first 6 months was present in 36.7% of non-treated eyes. Our data indicate that in those eyes, treatment could be considered since VA outcomes were worse in eyes which continued to be non-treated compared to eyes which were treated.

Eyes that were treated intensively did not experience a VA gain in our study as reported before in RCTs [3, 4, 10–12]. This may be due to the ceiling effect when starting with good vision. In the whole cohort, an intensive anti-VEGF treatment on average led to better anatomical outcomes compared to no treatment, which may or may not lead to a better long-term vision. Randomized prospective studies are required and we eagerly await the results of the DRCR.net protocol V [13]. This RCT includes eyes with center-involving DME and good visual acuity (defined as a ≥ 20/25 Snellen equivalent, ≥ 79 letters) that receive (1) prompt focal/grid photocoagulation + deferred anti-VEGF, (2) observation + deferred anti-VEGF, or (3) prompt anti-VEGF therapy [13]. The primary outcome is set as VA loss of ≥ 5 letters after 2 years [13]. It is vital to know whether early treatment in DME patients with very good visual acuity leads to better long-term visual outcomes, since anti-VEGF treatment is not without ocular and systemic risk [17, 18], and cause high costs to healthcare system and patient [19].

Limitations of this study include its retrospective nature and the shortcomings of a real-world setting, especially the lack of defined treatment criteria among the study centers. Baseline characteristics between treated and untreated eyes were not well balanced, with unsurprisingly more severe cases in the treatment group. To account for this we included baseline characteristics as confounders in the statistical analyses. We were not able to report outcomes for untreated eyes with higher CST, since those patients tended to be treated in our real-world setting. Thus, our results might not be applicable for patients with CST > 400 µm. We did not have information on the course of DME in the individual eyes before inclusion of the study, which might have also influenced the outcome results. Furthermore, we conducted multiple testing, which could have led to false-positive results.

This study shows, in a real-world setting, that the majority of eyes with DME and very good visual acuity maintain very good vision at 12 months whether the DME is treated or not. In the treated cohort, many anti-VEGF treatments at high cost to the patient and healthcare system were required to obtain small and clinically not relevant gains in VA and reduction in CST. This study, therefore, supports a close observation of eyes with DME and very good visual acuity at least until a one line drop in vision is observed, however, longer, randomized prospective studies are required.
